# A Simple Explicit Expression for the Flocculation Dynamics Modeling of Cohesive Sediment Based on Entropy Considerations

**DOI:** 10.3390/e20110845

**Published:** 2018-11-04

**Authors:** Zhongfan Zhu

**Affiliations:** College of Water Sciences, Beijing Normal University, Xinjiekouwai Street 19, Beijing 100875, China; zhuzhongfan1985@bnu.edu.cn; Tel.: +86-10-5880-2736

**Keywords:** entropy, Shannon entropy, Tsallis entropy, probability distribution, flocculation, cohesive sediment

## Abstract

The flocculation of cohesive sediment plays an important role in affecting morphological changes to coastal areas, to dredging operations in navigational canals, to sediment siltation in reservoirs and lakes, and to the variation of water quality in estuarine waters. Many studies have been conducted recently to formulate a turbulence-induced flocculation model (described by a characteristic floc size with respect to flocculation time) of cohesive sediment by virtue of theoretical analysis, numerical modeling, and/or experimental observation. However, a probability study to formulate the flocculation model is still lacking in the literature. The present study, therefore, aims to derive an explicit expression for the flocculation of cohesive sediment in a turbulent fluid environment based on two common entropy theories: Shannon entropy and Tsallis entropy. This study derives an explicit expression for the characteristic floc size, assumed to be a random variable, as a function of flocculation time by maximizing the entropy function subject to the constraint equation using a hypothesis regarding the cumulative distribution function of floc size. It was found that both the Shannon entropy and the Tsallis entropy theories lead to the same expression. Furthermore, the derived expression was tested with experimental data from the literature and the results were compared with those of existing deterministic models, showing that it has good agreement with the experimental data and that it has a better prediction accuracy for the logarithmic growth pattern of data in comparison to the other models, whereas, for the sigmoid growth pattern of experimental data, the model of Keyvani and Strom or Son and Hsu model could be the better choice for floc size prediction. Finally, the maximum capacity of floc size growth, a key parameter incorporated into this expression, was found to exhibit an empirical power relationship with the flow shear rate.

## 1. Introduction

Cohesive sediment, which is different from non-cohesive sediments such as sand, gravel, and cobbles, is a mixture of water, fine-grained sediments, such as silt, clay, and organic matter of diverse natures [1,2]. When cohesive sediment particles are transported in rivers, reservoirs, lakes, estuarines, and coastal waters, they continually flocculate to form flocs of different sizes due to small-scale, particle-particle interactions. On the other hand, some fragile and loose flocs may break into small flocs and/or primary particles (floc breakage or floc disaggregation) due to the flow shear [3,4,5]. Flocs are totally different from primary sediment particles in terms of their larger sizes, lower excess density, and higher settling velocity in water [2,6]. Studying cohesive sediment flocculation in a turbulent flow environment is essential because it plays an important role in affecting the morphological changes to coastal areas, dredging operations in navigational canals, and sediment siltation in reservoirs and lakes [7,8]. Since some pollutants (such as heavy metals) and nutrients are absorbed on the surfaces of cohesive sediment particles due to the electrochemical attraction of clay particles and/or organic matter contained in the sediment, the flocculation of cohesive sediment is also a vital element in investigating the variation of water quality and ecosystem function in some waters such as lakes and estuarine and coastal waters, which contain an abundance of cohesive sediment [9,10].

The turbulence-induced flocculation of cohesive sediment and other particles (such as polystyrene/latex particles) in a fluid environment has been investigated by several researchers in many research fields, including chemical and environment engineering, oceanography, and river and estuarine mechanics (e.g., References [11,12,13,14,15,16,17,18,19,20,21,22,23]). Most studies regarding turbulence-induced particle flocculation have focused on two main aspects: (1) the floc properties (mainly characterized by floc size or floc structure) at the steady or equilibrium states, in which the property parameters reach constant values; and (2) temporal variations of the size distribution and the structural and morphological properties of the flocs (commonly characterized by different fractal dimensions of the flocs) during the flocculation/aggregation process.

Some experimental works have been performed to investigate the median value of the size distribution of the flocs at the steady state of flocculation with respect to various flow shear conditions (e.g., References [8,14,24,25,26,27]. These studies reported that the median floc size decreases as the flow shear stress increases. Furthermore, a power relationship function was commonly adopted to describe this dependence: the median size = c* (flow shear parameter) −γ, where c and γ are two positive constants. The c is the floc strength, which strongly depends on the method used to measure the floc size, while the γ is the stable floc size exponent depending on the breakage mechanisms (erosion or fracture) for flocs smaller or larger than the smallest eddy (i.e., Kolmogorov microscale) in the turbulent flow [28,29]. Some studies have focused on the structural and morphological properties of the flocs at the steady state of flocculation with respect to various flow shear conditions (e.g., References [6,17,25,30]). For example, Stone and Krishnappan [30] showed that particle boundaries become more convoluted and the shape of larger particles are more irregular at higher levels of flow shear stress, whereas Zhu et al. [6] reported that with increasing flow shear rates, the flocs become less elongated and their boundary lines become tighter and more regular.

The time evolution of the size distribution of the flocs during the turbulence-induced flocculation has been investigated by some researchers using experimental observational techniques or numerical modeling methods (e.g., References [14,16,24,31,32]). Some experimental works have reported a typical trend of floc size with respect to flocculation time: the median value of the size distribution of the floc population grows rapidly with time at the beginning of the flocculation experiment. This is because the flow shear increases the collision and adhesion between primary particles, producing some large flocs in the system [24,25,31]. However, as flocculation progresses further, the rapidity with which the median floc size increases with time begins to decline (that is, the floc size experiences a slowly increasing process with flocculation time) because large flocs possess a fragile and loose structure and are susceptible to breakage induced by the flow shear [12,14]. Finally, the median floc size reaches a steady or equilibrium state after a long flocculation time as a result of a dynamic balance between the floc growth and the floc breakage, which are both caused by the flow shear [16,33]. On the other hand, some authors have developed theoretical and/or numerical models to describe the temporal evolution of the size distribution of a floc population, among which a simplified Lagrangian flocculation model is the focus of some works [1,3,34,35]. The earliest form of this model was developed by Winterwerp [3] with a linear combination of the formulations for the floc aggregation and the floc breakage process due to the flow turbulence, with a constant fractal dimension of flocs during flocculation. This model primarily tracks the time evolution of a characteristic floc size (commonly the median value of the size distribution of flocs) during flocculation, and its prediction accuracy is further improved by some authors by virtue of modifying some key parameters that have been incorporated into the model [1,34]. Additionally, there have also been some experimental studies to demonstrate the temporal variations of structural and geometric properties of flocs during the turbulence-induced flocculation process (e.g., References [17,25,36]).

This study focuses on the temporal evolution of the characteristic floc size during flocculation. To the best of my knowledge, most of the studies regarding turbulence-induced flocculation have been performed theoretically, with deterministic approaches. However, a probability approach to investigate the flocculation process is still lacking in the literature. For the last two decades, the probabilistic treatment of hydraulic engineering problems based on entropy theory has gained the attention of some researchers [37]. For example, entropy-based studies have been conducted by many researchers to investigate the velocity distribution (e.g., References [38,39,40,41]), sediment concentration [42,43,44], and shear stress distribution [45,46] in open channels. Recent work on the application of the entropy theory in a classical hydraulic engineering problem can be found in the study of Singh et al. [37]. It should be noted that the entropy-based probability method might also be an easy and applicable tool for predictions in some hydraulic problems, even though the traditional deterministic approaches have provided some physical insights.

This study attempts to derive an entropy-based expression for the temporal evolution of the characteristic floc size during flocculation by using two known entropy theories: Shannon entropy and Tsallis entropy. Section 2 and Section 3 derive the characteristic floc size as a function of flocculation time using these two entropy theories. The derived expression is tested against experimental observation data in Section 4, and Section 5 contains a comparison of the expression with some developed deterministic models, as well as a discussion of the maximum capacity of floc size growth, a key parameter that has been incorporated into the expression. Finally, Section 6 presents the concluding remarks.

## 2. Shannon Entropy Theory for Flocculation Expression

The determination of the flocculation process of cohesive sediment using the Shannon entropy theory entails the following steps: (1) the definition of the Shannon entropy; (2) the specification of constraints; (3) the maximization of entropy; (4) the determination of the Lagrange multiplier; (5) the hypothesis regarding cumulative probability distribution; and (6) the derivation of the flocculation expression.

### 2.1. Definition of Shannon Entropy

Considering entropy as a measure of information and, therefore, of uncertainty, Shannon [47] formulated what is referred to as the Shannon entropy theory. The Shannon entropy quantitatively measures the mean uncertainty associated with a probability distribution of a random variable. Consider the characteristic floc size D, during the flocculation process as a continuous random variable [48,49]. The objective of this study is to derive the characteristic floc size as a function of flocculation time, t. For simplicity, we let the excess floc size, $\hat{D}\left(t\right)$, be defined as $\hat{D}\left(t\right)=D_{\infty }-D\left(t\right)$, where $D_{\infty }$ is the steady or equilibrium state value of the floc size. Therefore, at the beginning of flocculation (t=0), there is $\hat{D}\left(0\right)=D_{\infty }-D_{0}$, where $D_{0}$ is the median size of primary particles, whereas after a long time of flocculation (t→∞), there is a relation: $\hat{D}\left(t\right)=0$. Thus, the excess floc size $\hat{D}\left(t\right)$ will vary from 0 to $\hat{D}\left(0\right)$. For the probability density function of the excess floc size $\hat{D}\left(t\right)$, $f(\hat{D})$, the Shannon entropy, denoted by $H_{S}(\hat{D})$, can be expressed in the general form as

(1) $$H_{S}(\hat{D})=-\int_{0}^{D_{\infty }-D_{0}}f(\hat{D})\left(\ln f(\hat{D})\right)d\hat{D}\;$$

Theoretically, the Shannon entropy is at a maximum when the probability density function is uniform within its limits. Equation (1) expresses a measure of uncertainty of $f(\hat{D})$ or the average information content of sample $\hat{D}$.

### 2.2. Specification of Constraint

The total probability law must be satisfied for the probability density function $f(\hat{D})$. Therefore, the constraint equation can be written as

(2) $$\int_{0}^{D_{\infty }-D_{0}}f(\hat{D})d\hat{D}=1\;$$

### 2.3. Maximization of Entropy 

To derive the specific form of $f(\hat{D})$, we adopted the principle of maximum entropy developed by Jaynes [50,51,52]. This principle states that the least biased probability of $\hat{D}$, $f(\hat{D})$, will be the one that will maximize $H(\hat{D})$ given by Equation (1), subject to the given information on $\hat{D}$ expressed as a constraint equation. Such a probability distribution is yielded by the maximization of the Shannon entropy. To that end, the method of the Euler–Lagrange calculus of variation is used [37]. The Lagrangian function L can be written as follows:

(3) $$L=-f(\hat{D})\ln f(\hat{D})+\left(1-\lambda_{0}\right)f(\hat{D})\;$$

where $\lambda_{0}$ is the zeroth Lagrange multiplier.

Differentiating Equation (3) with respect to $f(\hat{D})$ and equating the derivative to zero, the probability density function $f(\hat{D})$ of the excess floc size $\hat{D}$ is given as

(4) $$f(\hat{D})=\exp(-\lambda_{0})\;$$

Therefore, in the cumulative distribution function (CDF), $f(\hat{D})$ of $\hat{D}$ is obtained by using Equation (4) as follows:

(5) $$F(\hat{D})=P\left(d\leq \hat{D}\right)=\exp(-\lambda_{0})\hat{D}\;$$

Both the probability density function and the cumulative distribution function depend on the value of the zeroth Lagrange multiplier $\lambda_{0}$.

### 2.4. Determination of the Lagrange Multiplier

Inserting Equation (4) into the constraint equation (Equation (2)) leads to the following relation:

(6) $$\int_{0}^{D_{\infty }-D_{0}}\exp(-\lambda_{0})d\hat{D}=1\Rightarrow f(\hat{D})=\frac{1}{D_{\infty }-D_{0}}\;$$

The combination of Equations (4) and (6) gives the following:

(7) $$\lambda_{0}=\ln\left(D_{\infty }-D_{0}\right)\;$$

The value of the Lagrange multiplier $\lambda_{0}$ can be obtained as long as the values of $D_{0}$ and $D_{\infty }$ are known from the observational data. 

### 2.5. Hypothesis on the Cumulative Distribution Function 

To derive the temporal evolution of the excess floc size $\hat{D}$ in the real (space) domain, an equation connecting the probability domain to the space domain is required [37]; therefore, a hypothesis on the CDF of the excess floc size $\hat{D}$ is made so that the hypothesized CDF can reflect the characteristic of $\hat{D}$.

**Figure 1:**
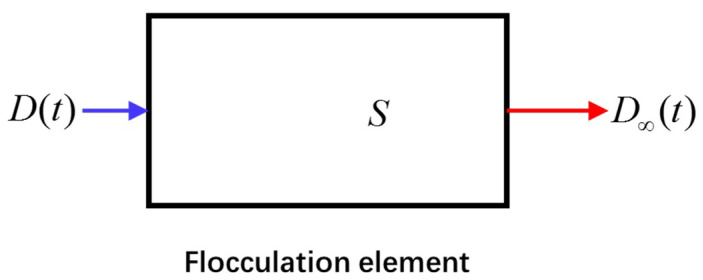
The flocculation element with floc size growth D(t) = floc size entering the flocculation element, $D_{\infty }\left(t\right)$ = floc size exiting the element, and S = the capacity of floc size growth.

Consider a simple flocculation element as shown in Figure 1. At the beginning of flocculation, some primary particles collide and adhere in small flocs due to the eddy motion of the turbulent flow, and the floc size D(t) increases significantly [18,19,25]. Whereas after a certain flocculation time, those formed fragile and loose flocs easily undergo a breakup due to the flow shear; therefore, the floc size growth begins to decline [12,14]. Let the floc size exiting the flocculation element be denoted as $D_{\infty }$ which approximately equals the steady state of the floc size. The flocculation element will have a maximum capacity of floc size growth, denoted by S (its unit should be in m*s). If we define the cumulative floc size growth as J (its unit should also be in m*s), then 0≤J≤S for the flocculation element. The continuity equation for the flocculation element, as shown in Figure 1, can be expressed as

(8) $$\frac{dJ}{dt}=D_{\infty }-D\left(t\right);\;\mathrm{or}\;J\left(t\right)=D_{\infty }t-\int_{0}^{t}D\left(t\right)dt\;$$

It is hypothesized that the cumulative distribution function F(D) of the floc size can be defined as the ratio of the cumulative floc size growth to the maximum capacity of the floc size growth or maximum potential floc size growth, S:

(9) $$F(D)=\frac{J}{S}\;$$

Here S has the same units as J. In Equation (9), it is implied that all of the values of the cumulative floc size growth are equally likely. A similar hypothesis has been employed by Chiu [53] and Kumbhakar and Ghoshal [41] for deriving a one-dimensional velocity distribution in open channels, by Chiu et al. [42] and Kumbhakar et al. [44] for deriving the sediment concentration profiles, and by Khozani and Bonakdari [54] for deriving the shear stress distribution in open channels. As Singh [55] showed, even if the above hypothesis is not strictly valid, it will not greatly influence the results because it merely allows the entropy theory to lead to the equation for floc size growth that is desired.

The differentiation of Equation (9) gives

(10) $$\frac{dF(D)}{dD}=f\left(D\right)=\frac{1}{S}\frac{dJ}{dD}\;$$

### 2.6. Derivation of the Flocculation Process

Combining Equations (6), (8), and (10) yields

(11) $$\frac{1}{D_{\infty }-D_{0}}\frac{dD}{dt}=\frac{1}{S}\left(D_{\infty }-D\right)\;$$

Integrating Equation (11) and using the initial condition: $D=D_{0}$ at t=0, we obtain

(12) $$D\left(t\right)=D_{\infty }-\left(D_{\infty }-D_{0}\right)\exp\left(-\frac{D_{\infty }-D_{0}}{S}t\right)\;$$

Substituting Equation (12) into Equation (8), the cumulative floc size growth J is written as

(13) $$J=S\left[1-\exp\left(-\frac{D_{\infty }-D_{0}}{S}t\right)\right]\;$$

Inserting Equation (13) in Equation (9) yields the cumulative distribution function F(D) of the floc size as

(14) $$F\left(D\right)=\exp\left(-\frac{D_{\infty }-D_{0}}{S}t\right)\;$$

Finally, the entropy of the probability distribution of the floc size $H_{S}(D)$ is obtained by substituting Equation (6) into Equation (1) as follows:

(15) $$H_{S}\left(D\right)=\ln\left(D_{\infty }-D_{0}\right)\;$$

Equation (15) states that the uncertainty of the floc size depends on the initial floc size value $D_{0}$ and the steady state value $D_{\infty }$.

## 3. Tsallis Entropy Theory for the Flocculation Model

The application of the Tsallis entropy theory into the derivation of the floc size as a function of flocculation time D(t) contains the same procedure as the Shannon entropy. 

If the floc size D(t) is considered as a continuous random variable with a probability function defined as f(D), another entropy function that has been termed as Tsallis entropy, $H_{T}(D)$, which was proposed by Tsallis [56] as a generalized form of the Shannon entropy, can be written as follows:

(16) $$H_{T}\left(D\right)=\frac{1}{m-1}\left\{1-\int_{D_{0}}^{D_{\infty }}{\left[f\left(D\right)\right]}^{m}dD\right\}\;$$

where m is a real number not equal to 1. The Tsallis entropy is a non-extensive entropy that reduces to the Shannon entropy if the exponent m→1 in Equation (16). For any m, it takes its maximum value in the case of equiprobability, and this entropy function reaches its maximum (concave function) if m 0 and its minimum (convex function) if m 0 for a certain value of m [57].

Similar to the Shannon entropy method, the constraint equation that f(D) must satisfy becomes

(17) $$\int_{D_{0}}^{D_{\infty }}f(D)dD=1\;$$

Using the principle of maximum entropy, the Lagrangian function $L^{’}$ for the Tsallis entropy can be written as follows:

(18) $$L^{’}=\frac{1}{m-1}\left\{1-\int_{D_{0}}^{D_{\infty }}{\left[f\left(D\right)\right]}^{m}dD\right\}+\lambda_{0}^{’}\left[\int_{D_{0}}^{D_{\infty }}f(D)dD-1\right]\;$$

where $\lambda_{0}^{’}$ is the zeroth Lagrange multiplier. Differentiating Equation (18) with respect to f(D) and equating the derivative to zero, the probability density function f(D) of the floc size is obtained as

(19) $$f(D)={\left[\frac{m-1}{m}\left(\frac{1}{m-1}+\lambda_{0}^{’}\right)\right]}^{\frac{1}{m-1}}\;$$

Substituting Equation (19) into Equation (17), we get

(20) $$f(D)=\frac{1}{D_{\infty }-D_{0}},\;\mathrm{and}\;\lambda_{0}^{’}\;=\;\frac{m}{m-1}{\left(\frac{1}{D_{\infty }-D_{0}}\right)}^{m-1}-\frac{1}{m-1}\;$$

Combining Equations (8), (10), and (20) yields: $1/\left(D_{\infty }-D_{0}\right)dD/dt=\left(D_{\infty }-D\right)/S$, which is Equation (11). In the same way as the Shannon entropy method, by integrating this equation and using the initial condition $D=D_{0}$ at t=0, we obtain the function of D(t) as Equation (12). Similarly, the cumulative distribution function F(D) is also derived as Equation (14). 

Inserting Equation (20) into Equation (16), we obtain the Tsallis entropy of the probability density function of the floc size as

(21) $$H_{T}\left(D\right)=\frac{1}{m-1}\left[\left(D_{\infty }-D_{0}\right)-{\left(D_{\infty }-D_{0}\right)}^{1\;-\;m}\right],$$

which depends on three parameters: the initial floc size value $D_{0}$, the steady state value $D_{\infty }$, and the parameter m.

It can be seen that both the Shannon entropy and the Tsallis entropy produce the same analytical expression (Equation (12)) that describes the temporal evolution of floc size during the turbulence-induced flocculation process. We also need to point out that the proposed model (Equation (12)) refers to a monodisperse distribution system, as the heterodisperse characteristic of aggregates were not considered at the presented modeling.

## 4. Results

Thirty-three experimental data sets regarding the floc size with respect to flocculation time in the published literature were collected to test the validity of the entropy-based expression (Equation (12)) in this study. Table 1 presents the information on these collected experimental data. The first column number is the experimental data. The second column introduces the particle material: some adopted the sediment material, whereas some used the polystyrene/latex material, and the third column presents the apparatus for generating the turbulent flocculation environment. In the fourth column, ϕ is the particle volumetric concentration (it is equal to the volume of the primary particle divided by the volume of the particle-liquid mixture). G is the flow shear rate (its unit is 1/s), defined as $\sqrt{\varepsilon /\nu }$, where ε is the turbulent dissipation rate of the turbulent flow and ν is the kinematic viscosity of the fluid, as adopted by many studies [12,14,16,25,29]. The measured size of the primary particles and the floc size at the steady state of flocculation are shown in the fifth and sixth columns, respectively, and the data source is identified in the last column. The criteria used to take experiments from the literature for modeling validation are that the selected data sets cover different flocculation materials (sediment or polystyrene/latex particle), different flocculation environments (Couette-flow system or baffled stirred tank), and various flow shear conditions (the low flow turbulent condition, for example, G = 0.45, 0.75, 0.96, 2.4 $s^{-1}$; the moderate flow turbulent condition, for example, G = 19.4, 25, 37, 50 $s^{-1}$; the strong turbulent condition, for example, G = 100, 150, 20, 246 $s^{-1}$).

To evaluate the performance of the derived entropy-based flocculation expression with experimental observation data and some deterministic models, an error analysis is performed by computing the correlation coefficient $R^{2}$ between the modeled and the observed data, the relative bias (*RBIAS*) between the modeled and the observed data, defined as *RBIAS* = $\frac{1}{N}\sum_{i=1}^{N}\left|\frac{m_{i}-o_{i}}{o_{i}}\right|$, and the root-mean-square error (*RMSE*), defined as *RMSE* = $\sqrt{\frac{1}{N}\sum_{i=1}^{N}{\left(m_{i}-o_{i}\right)}^{2}}$, where m and o are the modeled and observed points, respectively, and N is the number of observed points. The goodness of fit increases as the $R^{2}$ value increases and both the *RBIAS* and *RMSE* values decrease.

**Figure 2:**
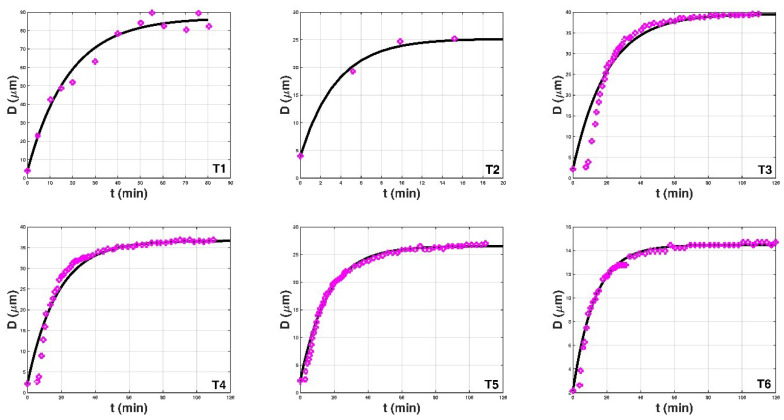

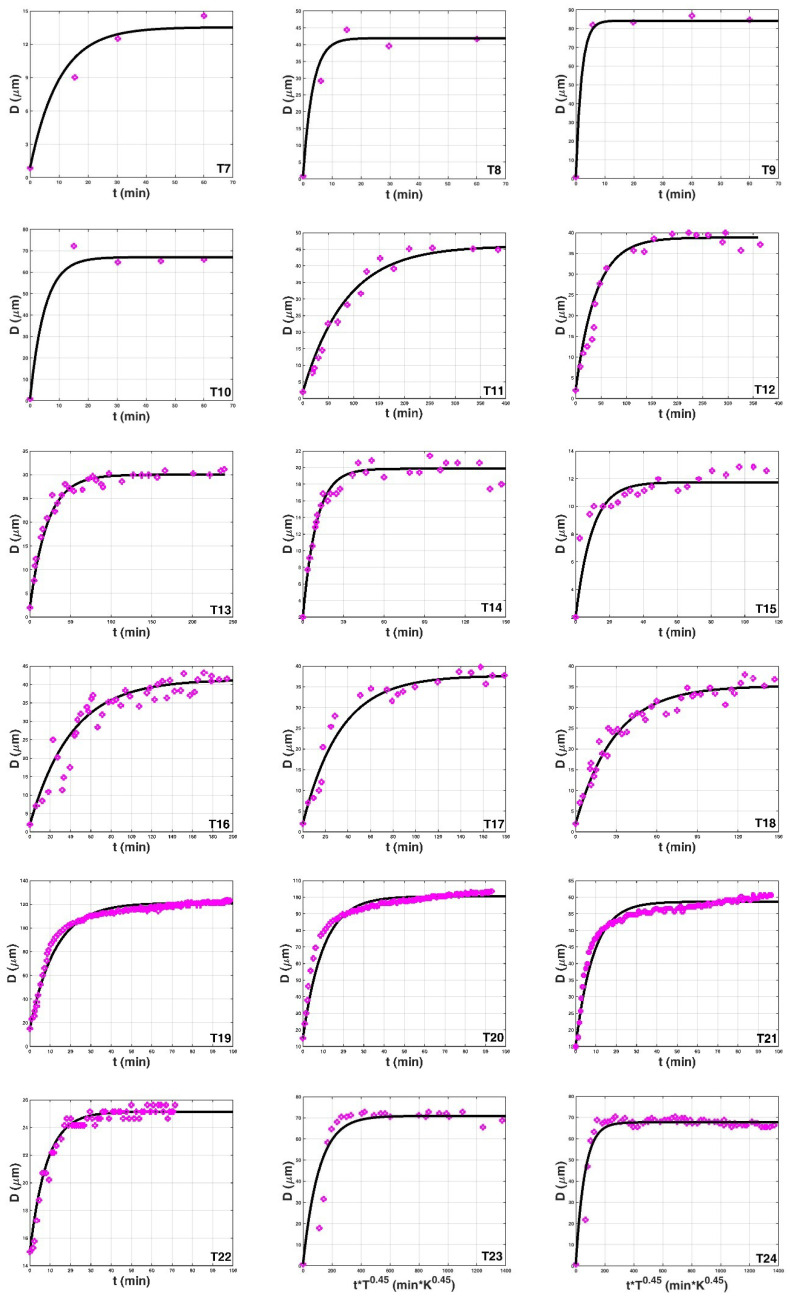

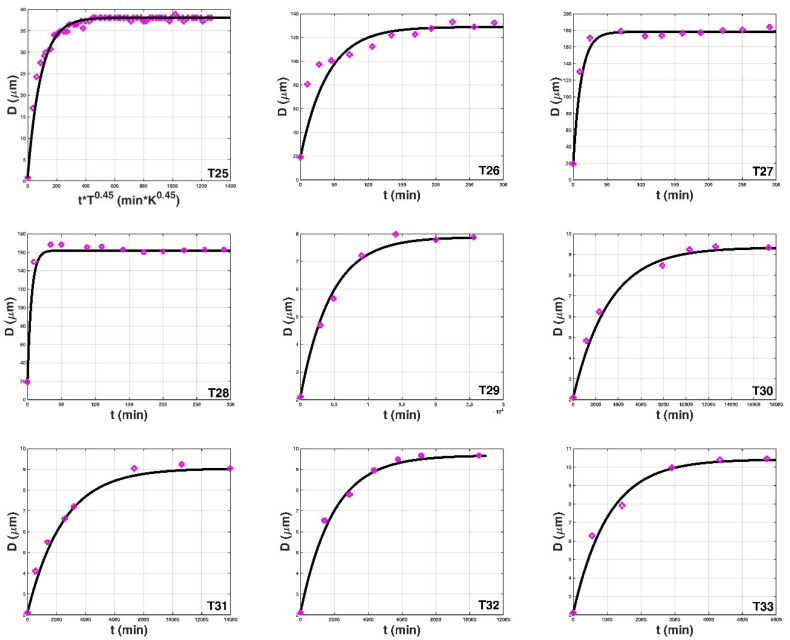
The comparison of the proposed entropy-based expression (Equation (12)) with thirty-three experimental data sets from the literature. In each figure, the magenta circles denote the measured data and the black line represents the proposed expression. For the cases of T23, T24, and T25 from Selomulya et al. [60], the horizontal axis is not the flocculation time t but a quantity $t*T^{0.45}$, where T is the absolute temperature in the flocculation time (the unit is Kelvin), and we cannot obtain the value of the flocculation time from their paper.

Figure 2 shows the comparison of the proposed entropy-based model with the collected experimental data. Table 2 presents the comparison results. From the third, fourth, and fifth columns, it can be seen that there is a high $R^{2}$ value and low *RBIAS* and *RMSE* values for each case. Additionally, the entropy function values estimated by Equations (15) and (21) are also presented in the last columns. These results indicate that the proposed entropy-based model shows a good agreement with the experimental data.

## 5. Discussion

### 5.1. Comparison with the Deterministic Model

To further test the prediction accuracy of the proposed entropy-based expression, we compare it with some deterministic models. There have been three main simplified Lagrangian flocculation models: Winterwerp [3], Son and Hsu [1], and Son and Hsu [34]. Table 3 list these models. c and $\rho_{s}$ are the mass concentration and the density of primary particles, respectively; $k_{A}$ is the dimensionless coefficient for floc aggregation; $k_{B}$ is the dimensionless coefficient for floc breakage; $d_{f}$ and F are the fractal dimension and yield strength of the flocs, respectively; μ is the dynamic viscosity of the fluid; α and β are two coefficients; and B is a coefficient representing the cohesive force between the primary particles.

In the study of Son and Hsu [34], a comparison among the Winterwerp model, the Son and Hsu [1] model and the Son and Hsu [34] model were conducted with the experimental results of Burban et al. [58] and an experimental data set from Biggs and Lant [14]. To simplify the problem, we attempted to compare the proposed entropy-based model (Equation (12)) with the Winterwerp model, the Son and Hsu (2008) model, and the Son and Hsu (2009) model for the experimental data in this study. Table 4 presents the calculated $R^{2}$, *RBIAS*, and *RMSE* values for these real cases. It can be observed that the proposed model has the highest $R^{2}$ value and the lowest *RBIAS* and *RMSE* values in comparison with the other three models for all of the real cases. For the case of ϕ = 1.66 × 10^−3^ from Burban et al. [58], the proposed model has the highest $R^{2}$ value and the lowest *RBIAS* value compared with the other models, whereas the model of Son and Hsu (2008) yields a lower *RMSE* value than the proposed model, which may be because of the very limited experimental data. Hence, this study shows the potential of the Shannon entropy together with the principle of maximum entropy to predict the temporal evolution of floc size during flocculation.

**Figure 3:**
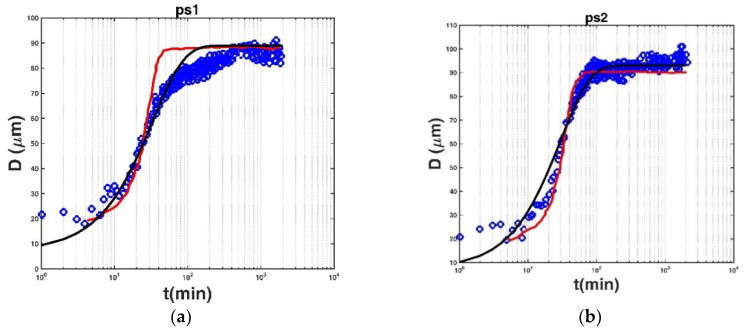

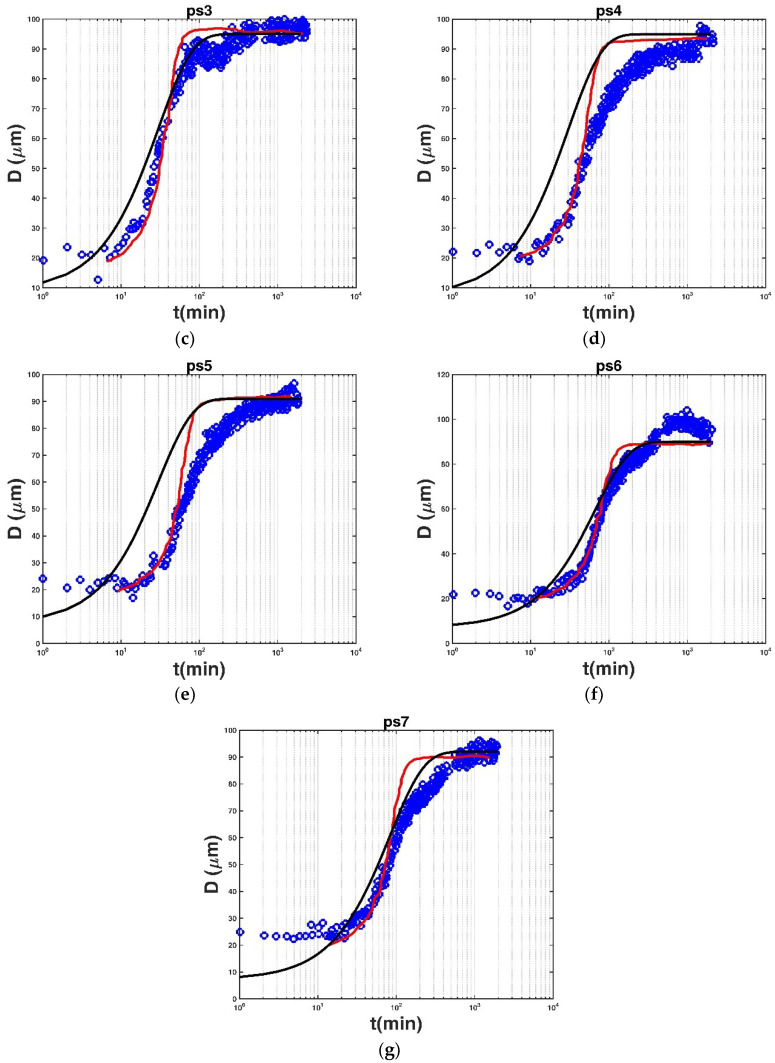
The comparison of the entropy-based expression with the model of Keyvani and Strom for measured data in each of the cycles: (**a**) ps1, (**b**) ps2, (**c**) ps3, (**d**) ps4, (**e**) ps5, (**f**) ps6, and (**g**) ps7 in the work of Keyvani and Strom [33] (ps was referred to as a “prior shear” case, corresponding to the cycle order of the high and low turbulent shear, in their paper). The blue circle denotes the measured data, the black line represents the entropy-based expression, and the red line shows the model of Keyvani and Strom.

In the work of Keyvani and Strom [33], the effects of seven cycles of high and low turbulent shear on mud floc growth pattern and equilibrium size were investigated through a laboratory study. The measured temporal variation of the mean floc size in each of the seven cycles was modelled using the Winterwerp model after calibrating the collision and breakup efficiency coefficients for each cycle in their paper. Here we attempted to compare the entropy-based expression (Equation (12)) and the model of Keyvani and Strom for these measured data, as shown in Figure 3. Note that the horizontal axis in these figures refers to the logarithmic coordinates. Most of the measured data show a sigmoid growth pattern of floc size with flocculation time. For the sigmoid growth curve of floc size with flocculation time, it could be found from Figure 3 that the entropy-based expression (Equation (12)) did not exhibit a satisfactory fitting result for these measured data (especially in Figure 3d,e), however, the model of Keyvani and Strom presents a typical sigmoid growth property and shows a better prediction accuracy in comparison to Equation (12). In Figure 2, most of the measured data show a logarithmic growth pattern of floc size with flocculation time, and the entropy-based expression has provided a good fitting result for them. This is because this expression is based on the assumption that the cumulative distribution of floc size can be defined as the ratio of the cumulative floc size growth to the maximum potential floc size growth, and this leads to the logarithmic result. It could be concluded that the entropy-based expression developed in this study can fit well for the logarithmic growth pattern of floc size, whereas for the sigmoid growth pattern of floc size, the model of Keyvani and the Strom or Son and Hsu (2009) model could be the better choice for floc size prediction. This limitation of the developed entropy-based expression could be worthy of further investigation in future research.

### 5.2. Estimation of the Key Parameter

**Figure 4:**
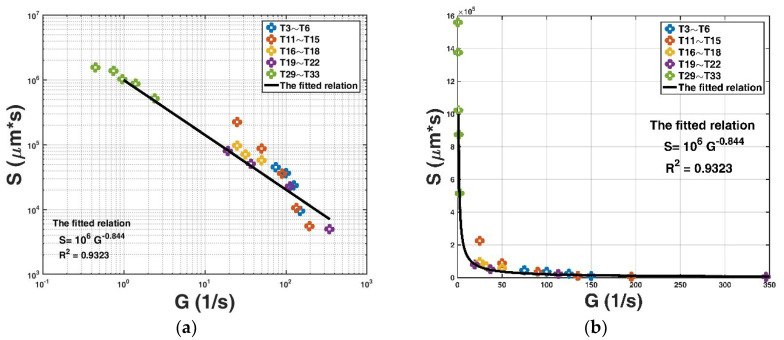
The fitted parameter value S in the proposed entropy-based model with respect to different flow shear rate G values for the collected experimental data in the log-log space (**a**) and the normal space (**b**).

**Figure 5:**
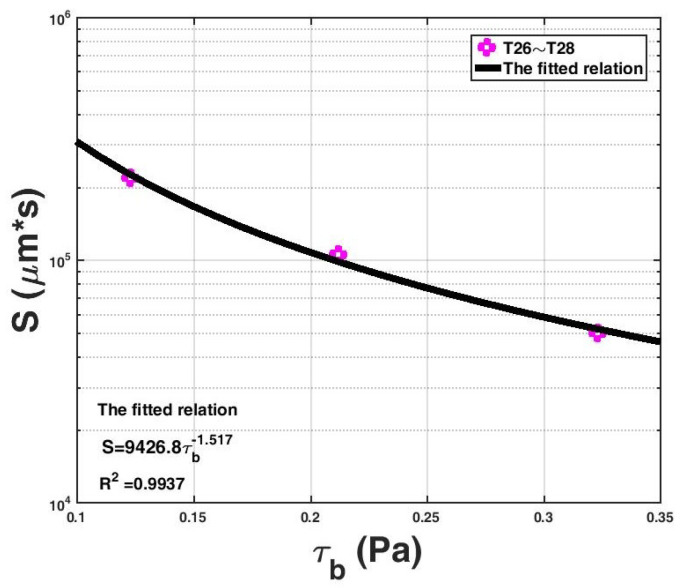
The fitted parameter value S in the proposed entropy-based model with respect to the different bed shear stress $\tau_{b}$ values for the experimental data from Stone and Krishnappan [30].

The key parameter that was incorporated into the expression (Equation (12)) is the capacity for floc size growth in the flocculation system S. Here, we compare the fitted values of S with the flow shear rate G for all experimental data (T1–T33), as shown in Figure 4, except for T1, T2, T7–T10, and T23–T25. The reason for these exceptions is that both studies of Burban et al. [58] (that is, T1–T2) and Spicer and Pratsinis [59] (that is, T7–T10) adopted the fixed G values, and the value of the absolute temperature T is not available in the study of Selomulya et al. [60] (that is, T23–T25); therefore, the calculated S does not have the same unit as the other real cases. It should be noted that in the study of Stone and Krishnappan [30] (that is T26–T28), only the bed shear stress $\tau_{b}$ in the turbulent-generating equipment is provided, and the flow shear rate G cannot be calculated; thus, Figure 5 shows the calculated S values with respect to different bed shear stresses for T26–T28. 

It can be seen from Figure 4 that there is a fitting relation between S and G as follows: $S=10^{6}G^{-0.844}$, with a very high coefficient of determination $R^{2}$ reaching 0.93. From Figure 5, there is a similar fitting relation between S and $\tau_{b}$ as follows: $S=9426.8\tau_{b}{}^{-1.517}$, with a coefficient of determination $R^{2}$ reaching 0.9937. This implies that as the flow shear condition intensifies, the capacity for floc size growth in the flocculation system decreases. This is because the floc breakage caused by the increasing flow shear plays an increasingly important role in the flocculation process. 

Substituting the aforementioned mathematical relation into Equation (12) leads to the final expression for floc size D as a function of flocculation time t as follows:

(22) $$D\left(t\right)=D_{\infty }-\left(D_{\infty }-D_{0}\right)\exp\left[-10^{-6}\left(D_{\infty }-D_{0}\right)G^{0.844}t\right]\;$$

for the collected experimental data, except for Stone and Krishnappan [30], whereas for the experimental data of Stone and Krishnappan [30], the expression has the following form: $D\left(t\right)=D_{\infty }-\left(D_{\infty }-D_{0}\right)\exp\left[-10^{-4}\left(D_{\infty }-D_{0}\right)\tau_{b}^{1.517}t\right]$.

**Figure 6:**
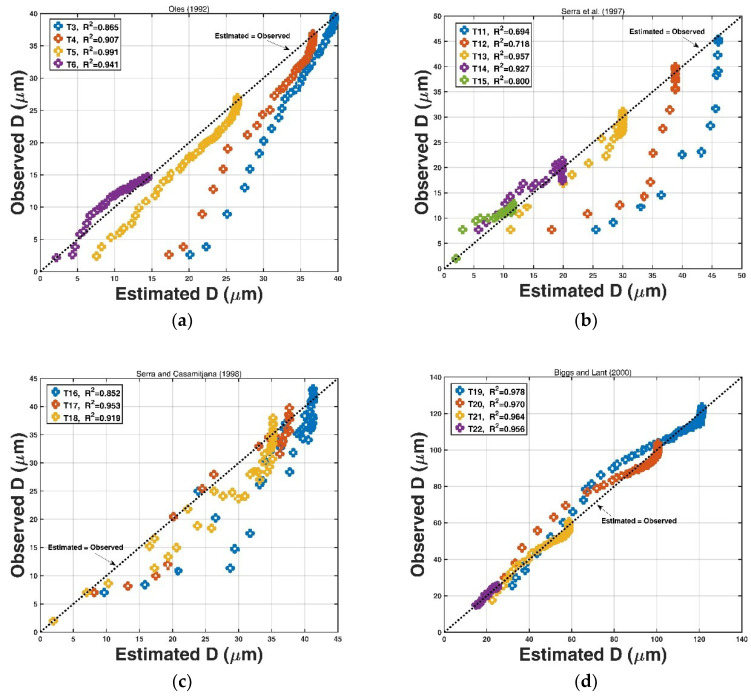

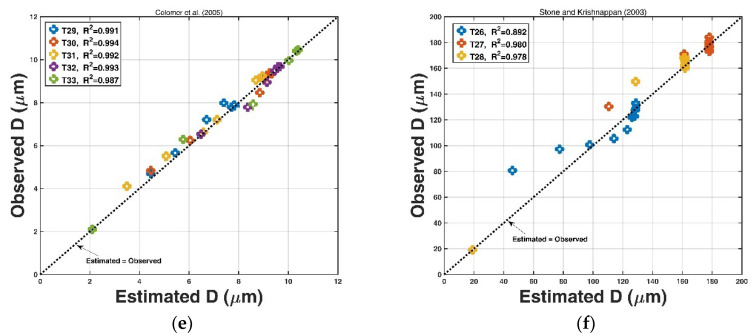
The comparison between the observed floc size D and the estimated floc size D using Equation (22) for the experimental data from (**a**) Oles [24], (**b**) Serra et al. [12], (**c**) Serra and Casamitjana [31], (**d**) Biggs and Lant [14], (**e**) Colomer et al. [61], and (**f**) Stone and Krishnappan [30].

We estimated the floc size D using Equation (22) and the aforementioned other expression, and compared them with the collected experimental data, as shown in Figure 6. It can be observed that there is a very high coefficient of determination $R^{2}$ between the estimated values and the observed ones for most data sets. Only four data sets (T3, T11, T12, and T16) have an $R^{2}$ value smaller than 0.90, and the reason may be that there is a data scattering perhaps due to the experimental measurement operation. These results imply that the proposed entropy-based expression has a good prediction ability for the temporal evolution of floc size during the turbulence-induced flocculation.

Similar to the Camp number P (this is equal to the product of flow shear rate and flocculation time, that is, P=Gt) defined in the research field of wastewater treatment [28], we define a new parameter P’ as $P’=G^{0.844}t$, and Equation (22) becomes $D\left(t\right)=D_{\infty }-\left(D_{\infty }-D_{0}\right)\exp\left[-10^{-6}\left(D_{\infty }-D_{0}\right)G^{0.844}t\right]=D_{\infty }-\left(D_{\infty }-D_{0}\right)\exp\left[-10^{-6}\left(D_{\infty }-D_{0}\right)P’\right]$. The floc size is a monotonic increasing function of the new parameter P’: it firstly undergoes a rapidly increasing process, and then a slowly increasing process before approaching a final steady state. When $D_{0}\ll D_{\infty }$, the floc size approximately reaches the steady state if P’ is larger than $3\;\times 10^{6}/D_{\infty }$. It is easy and applicable to adopt Equation (22) to predict the floc size during flocculation as long as the values of $D_{0}$, $D_{\infty }$, and G are given. Equation (22) provides a new method for flocculation dynamic modeling based on entropy considerations. It has a simple mathematical form, contains fewer parameter inputs compared with other existing deterministic models, and avoids an iteration calculation required in other models for the floc size estimation. This equation contains the effect of the flow shear on the floc breakage (as shown by Figure 4). However, some physical properties present in existing deterministic models are not incorporated into this equation. For example, the geometric structure of floc plays a role in both dynamic processes of floc growth and floc breakage. In the existing deterministic models, the fractal dimension of floc $d_{f}$ has been adopted to describe this property. However, the entropy-based expression does not contain this parameter.

## 6. Concluding Remarks

The following concluding remarks can be made from this study:

1. A simple explicit expression that describes the temporal evolution of the characteristic floc size during turbulence-induced flocculation was derived based on the entropy theory.
2. Both the Shannon entropy theory and the Tsallis entropy theory lead to the same expression for the function of floc size with respect to flocculation time.
3. The entropy-based expression was tested against the experimental data in the literature, and a good agreement was found.
4. The entropy-based expression was compared with other deterministic models, and it was found that the expression shows a better prediction accuracy for the logarithmic growth pattern of experimental data in comparison to the other models, whereas, for the sigmoid growth pattern of data, the model of Keyvani and Strom or the Son and Hsu (2009) model could be the better choice for floc size prediction.
5. The maximum capacity of floc size growth, a key parameter that was incorporated into the expression, exhibits an empirical power-law relation with the flow shear rate. As the flow shear condition intensifies, the capacity for floc size growth in the flocculation system decreases. This is because the floc breakage caused by the increasing flow shear plays an increasingly important role in the flocculation process.

## Figures and Tables

**Table 1:** The information on the collected experimental data in the literature.

Experimental Data Number	Experimental Material	Turbulence-Generating Environment	Flow Shear Condition	$D_{0}\;(\mu m)$	$D_{\infty }\;(\mu m)$	Data Source
T1	Detroit river sediment	Couette-flow chamber	ϕ = 1.04 × 10^−4^; *G* = 200 $s^{-1}$	4	87	Burban et al. [58]
T2			ϕ = 1.66 × 10^−3^; *G* = 200 $s^{-1}$	4	25.21	
T3	Polystyrene latex	Couette-flow system formed by two cylinders	ϕ = 5 × 10^−5^; *G* = 75 $s^{-1}$	2.17	39.54	Oles [24]
T4			ϕ = 5 × 10^−5^; *G* = 100 $s^{-1}$	2.17	36.65	
T5			ϕ = 5 × 10^−5^; *G* = 125 $s^{-1}$	2.17	26.52	
T6			ϕ = 5 × 10^−5^; *G* = 150 $s^{-1}$	2.17	14.47	
T7	Polystyrene particle	Baffled stirred tank	ϕ = 2.10 × 10^−5^; *G* = 63 s^−1^ Alum concentration: 4.3 mg/L	0.87	13.54	Spicer and Pratsinis [59]
T8			ϕ = 2.10 × 10^−5^; *G* = 63 s^−1^ Alum concentration: 10.7 mg/L	0.87	41.90	
T9			ϕ = 2.10 × 10^−5^; *G* = 63 s^−1^ Alum concentration: 32 mg/L	0.87	84.20	
T10			ϕ = 2.10 × 10^−5^; *G* = 95 s^−1^ Alum concentration: 32 mg/L	0.87	67.01	
T11	Latex particle	Couette-flow system	ϕ = 2.5 × 10^−5^; *G* = 25 $s^{-1}$	2	46.06	Serra et al. [12]
T12			ϕ = 2.5 × 10^−5^; *G* = 50 $s^{-1}$	2	38.84	
T13			ϕ = 2.5 × 10^−5^; *G* = 90 $s^{-1}$	2	30	
T14			ϕ = 2.5 × 10^−5^; *G* = 135 $s^{-1}$	2	19.87	
T15			ϕ = 2.5 × 10^−5^; *G* = 195 $s^{-1}$	2	11.74	
T16	Latex particle	Couette-flow system	ϕ = 5 × 10^−5^; *G* = 25 $s^{-1}$	2	41.36	Serra and Casamitjana [31]
T17			ϕ = 5 × 10^−5^; *G* = 32 $s^{-1}$	2	37.73	
T18			ϕ = 5 × 10^−5^; *G* = 50 $s^{-1}$	2	35.23	
T19	Activated sludge	Baffled batch vessel	ϕ = 5 × 10^−2^; *G* = 19.4 $s^{-1}$	15 ***	121.27	Biggs and Lant [20]
T20			ϕ = 5 × 10^−2^; *G* = 37 $s^{-1}$	15 ***	100.56	
T21			ϕ = 5 × 10^−2^; *G* = 113 $s^{-1}$	15 ***	58.66	
T22			ϕ = 5 × 10^−2^; *G* = 346 $s^{-1}$	15 ***	24.14	
T23	Polystyrene latex particle	Couette-flow system	ϕ = 3.76 × 10^−5^; *G* = 64 s^−1^	0.81	70.94	Selomulya et al. [60]
T24			ϕ = 3.76 × 10^−5^; *G* = 100 s^−1^	0.81	67.76	
T25			ϕ = 3.76 × 10^−5^; *G* = 246 s^−1^	0.81	38.07	
T26	Hay river sediment, Canada	Annular flume	Bed shear stress = 0.123 Pa	19.1	128.97	Stone and Krishnappan [30]
T27			Bed shear stress = 0.212 Pa	19.1	178.1	
T28			Bed shear stress = 0.323 Pa	19.1	161.84	
T29	Polystyrene latex particle	Flask shaking table	ϕ = 2 × 10^−5^; G = 0.45 $s^{-1}$	2.1	7.88	Colomer et al. [61]
T30			ϕ = 2 × 10^−5^; G = 0.75 $s^{-1}$	2.1	9.34	
T31			ϕ = 2 × 10^−5^; G = 0.96 $s^{-1}$	2.1	9.05	
T32			ϕ = 2 × 10^−5^; G = 1.41 $s^{-1}$	2.1	9.68	
T33			ϕ = 2 × 10^−5^; G = 2.4 $s^{-1}$	2.1	10.42	

The “***” symbol indicated that the measured size by Biggs and Lant [14] at the beginning of the flocculation experiment is actually the floc size of 15 microns rather than the size of the primary particle (the primary particle size is actually 4 microns).

**Table 2:** The comparison results of the proposed entropy-based expression with the collected experimental data in the literature.

Experimental Data Number	Data Source	Fitting Result			Entropy Function	
		$R^{2}\;$	*RBIAS*	*RMSE*	$H_{S}\left(D\right)\;$	$H_{T}\left(D\right)$ Assume *m* = 2
T1	Burban et al. [58]	0.975	0.054	4.170	4.419	82.988
T2		0.995	0.023	0.640	3.054	21.163
T3	Oles [24]	0.944	0.213	3.134	3.621	37.343
T4		0.948	0.160	2.341	3.540	34.451
T5		0.989	0.080	0.960	3.193	24.309
T6		0.982	0.044	0.512	2.510	12.219
T7	Spicer and Pratsinis [59]	0.962	0.076	1.053	2.539	12.591
T8		0.964	0.069	3.280	3.714	41.006
T9		0.999	0.014	1.511	4.423	83.318
T10		0.978	0.039	4.038	4.192	66.125
T11	Serra et al. [12]	0.981	0.118	2.445	3.786	44.037
T12		0.962	0.121	3.028	3.607	36.813
T13		0.976	0.045	1.261	3.332	27.964
T14		0.958	0.044	0.954	2.883	17.814
T15		0.850	0.076	1.078	2.276	9.637
T16	Serra and Casamitjana [31]	0.899	0.121	3.606	3.673	39.335
T17		0.952	0.104	2.600	3.576	35.702
T18		0.956	0.072	2.019	3.503	33.200
T19	Biggs and Lant [14]	0.980	0.027	3.403	4.666	106.261
T20		0.967	0.037	4.126	4.449	85.548
T21		0.960	0.036	2.087	3.776	43.637
T22		0.972	0.017	0.521	2.213	9.031
T23	Selomulya et al. [60]	0.845	0.124	7.607	4.250	70.116
T24		0.899	0.041	3.623	4.204	66.935
T25		0.979	0.019	1.106	3.618	37.233
T26	Stone and Krishnappan [30]	0.887	0.085	13.304	4.699	109.861
T27		0.974	0.035	8.351	5.069	158.994
T28		0.984	0.023	5.988	4.961	142.733
T29	Colomer et al. [61]	0.993	0.021	0.189	1.754	5.607
T30		0.992	0.035	0.286	1.980	7.102
T31		0.993	0.038	0.304	1.939	6.806
T32		0.994	0.021	0.220	2.026	7.448
T33		0.988	0.032	0.350	2.119	

**Table 3:** The three simplified Lagrangian flocculation models.

Model Name	Formulation
Winterwerp model	$\frac{dD}{dt}=\frac{c}{\rho_{s}}\frac{k_{A}}{d_{f}}GD_{0}^{F-3}D^{4-F}-\frac{k_{B}}{D_{0}d_{f}}\sqrt{\frac{\mu }{F}}G^{1.5}D^{2}\left(D-D_{0}\right)$
Son and Hsu (2008) model	$\frac{dD}{dt}=\frac{GD_{0}^{\beta }}{\beta \ln\frac{D}{D_{0}}+1}\left[\frac{c}{3\rho_{s}}k_{A}D_{0}^{d_{f}-3}D^{4-d_{f}-\beta }-\frac{k_{B}}{3D_{0}}\sqrt{\frac{\mu G}{F}}D^{2-\beta }\left(D-D_{0}\right)\right]$
Son and Hsu (2009) model	$\frac{dD}{dt}=\frac{GD_{0}^{\beta }}{\beta \ln\frac{D}{D_{0}}+1}\left[\frac{c}{3\rho_{s}}k_{A}D_{0}^{d_{f}-3}D^{4-d_{f}-\beta }-\frac{k_{B}}{3}\sqrt{\frac{\mu G}{B}}D_{0}^{\frac{d_{f}}{3}-1}D^{1-\beta +\frac{3-d_{f}}{3}}\left(D-D_{0}\right)\right]$

**Table 4:** The comparison of the present entropy-based model with the deterministic models for the experimental data.

References	Experimental Conditions	Fitting Effect											
		The Present Model			Winterwerp Model			Son and Hsu (2008) Model			Son and Hsu (2009) Model		
		$R^{2}\;$	*RBIAS*	*NRMSE*	$R^{2}\;$	*RBIAS*	*NRMSE*	$R^{2}\;$	*RBIAS*	*NRMSE*	$R^{2}\;$	*RBIAS*	*NRMSE*
Burban et al. [58]	ϕ = 1.04 × 10^−4^; G = 200 $s^{-1}$	0.98	0.054	4.170	0.83	0.282	19.860	0.86	0.255	17.587	0.90	0.190	12.942
	ϕ = 1.66 × 10^−3^; G = 200 $s^{-1}$	0.99	0.023	0.640	0.97	0.037	1.083	0.97	0.026	0.424	0.98	0.036	1.053
Biggs and Lant [14]	ϕ = 5 × 10^−2^; G = 19.4 $s^{-1}$	0.98	0.027	3.403	0.89	0.053	7.218	0.90	0.059	7.917	0.90	0.067	8.889
